# Hybrid algorithm for common solution of monotone inclusion problem and fixed point problem and applications to variational inequalities

**DOI:** 10.1186/s40064-016-2389-9

**Published:** 2016-06-21

**Authors:** Jingling Zhang, Nan Jiang

**Affiliations:** Department of Mathematics, Tianjin University, Tianjin, 300350 China

**Keywords:** Quasi-nonexpansive mappings, Inverse-strongly monotone mapping, Maximal monotone operator, Fixed point, 47H05, 47H09, 47H10

## Abstract

The aim of this paper is to investigate hybrid algorithm for a common zero point of the sum of two monotone operators which is also a fixed point of a family of countable quasi-nonexpansive mappings. We point out two incorrect proof in paper (Hecai in Fixed Point Theory Appl 2013:11, 2013). Further, we modify and generalize the results of Hecai’s paper, in which only a quasi-nonexpansive mapping was considered. In addition, two family of countable quasi-nonexpansive mappings with uniform closeness examples are provided to demonstrate our results. Finally, the results are applied to variational inequalities.

## Introduction and preliminaries

The monotone inclusion problem is to

$$\begin{aligned} {\text {find an}} \quad x\in H \quad {\text {such that}} \quad 0\in \sum _{i=1}^{m}A_ix, \end{aligned}$$

where *H* is a real Hilbert space with inner product $\langle \cdot , \cdot \rangle$ and $A_i$ are set-valued maximal monotone operators (Hui and Lizhi 2013). Such problem is very important in many areas, such as convex optimization and monotone variational inequalities, for instance. There is an extensive literature to approach the inclusion problem, all of which can essentially be divided into two classes according to the number of operators involved: single operator class $(m=1)$ and multiple operator class $(m\ge 2).$ The latter class can always be reduced to the case of $m=2$ via Spingarn’s method (Spingarn 1983). Based on a series of studies in the next decades, splitting methods for monotone operators were inspired and studied extensively. Splitting methods for linear equations were introduced by Peaceman and Rachford (1995) and Douglas and Rachford (1956). Extensions to nonlinear equations in Hilbert spaces were carried out by Kellogg (1969) and Lions and Mercier (1979). The central problem is to iteratively find a zero of the sum of two monotone operators *A* and *B* in a Hilbert space *H*. Splitting methods have recently received much attention due to the fact that many nonlinear problems arising in applied areas such as signal processing, image recovery and machine learning are mathematically modeled as a nonlinear operator equation (Shehu et al. 2016a, b; Shehu 2015). And the operator is decomposed into the sum of two nonlinear operators.

In this paper, we consider the problem of finding a solution for the following problem: find an *x* in the fixed point set of a family of countable quasi-nonexpansive mappings $S_n$ such that

$$\begin{aligned} x\in (A+B)^{-1}(0), \end{aligned}$$

where *A* and *B* are two monotone operators. The similar problem has been addressed by many authors in view of the applications in signal processing and image recovery; see, for example, Qin et al. (2010), Zhang (2012), Takahashi et al. (2010), Kamimura and Takahashi (2010) and the references therein.

Throughout this paper, we always assume that *H* is a real Hilbert space with the inner product $\langle \cdot ,\cdot \rangle$ and norm $\Vert \cdot \Vert$, respectively. Let *C* be a nonempty closed convex subset of $H,\;P_C$ be the metric projection from *H* onto *C*,  and $S: C \rightarrow C$ be a mapping. We use *F*(*S*) to denote the fixed point set of $S_n$ below, i.e., $F(S):= \{x \in C : x=Sx\}$. Recall that *S* is said to be nonexpansive if

$$\begin{aligned} \Vert Sx-Sy\Vert \le \Vert x-y\Vert , \quad \forall \quad x,y \in C. \end{aligned}$$

If *C* is a bounded closed and convex subset of *H*, then *F*(*S*) is nonempty closed and convex; see Browder (1976). *S* is said to be quasi-nonexpansive if $F(S)\ne \emptyset$ and

$$\begin{aligned} \Vert Sx-p\Vert \le \Vert x-p\Vert , \quad \forall \ x \in C, \quad p \in F(S). \end{aligned}$$

It is easy to see that nonexpansive mappings are Lipschitz continuous, however, the quasi-nonexpansive mapping is discontinuous on its domain generally. Indeed, the quasi-nonexpansive mapping is only continuous in its fixed point set.

Let $A : C\rightarrow H$ be a mapping. Recall that *A* is said to be monotone if

$$\begin{aligned} \langle Ax-Ay, x-y \rangle \ge 0, \quad \forall \ x,y \in C. \end{aligned}$$

*A* is said to be $\alpha$-strongly monotone if there exists a constant $\alpha >0$ such that

$$\begin{aligned} \langle Ax-Ay, x-y \rangle \ge \alpha \Vert x-y\Vert ^2, \quad \forall \ x,y \in C. \end{aligned}$$

*A* is said to be $\alpha$-inverse strongly monotone if there exists a constant $\alpha >0$ such that

$$\begin{aligned} \langle Ax-Ay, x-y \rangle \ge \alpha \Vert Ax-Ay\Vert ^2, \quad \forall \ x,y \in C. \end{aligned}$$

Notice that, a $\alpha$-inverse strongly monotone operator must be $\frac{1}{\alpha }$-Lipschitz continuous.

Recall that the classical variational inequality is to find an $x \in C$ such that

1 $$\begin{aligned} \langle Ax, y-x\rangle \ge 0, \quad \forall \ y \in C. \end{aligned}$$

In this paper, we use *VI*(*C*, *A*) to denote the solution set of (1). It is known that $x^*\in C$ is a solution to (1) if $x^*$ is a fixed point of the mapping $P_C(I-\lambda A)$, where $\lambda >0$ is a constant, *I* is the identity mapping, and $P_C$ is the metric projection from *H* onto *C*. Next we recall some well-known definitions.

### **Definition 1**

(*Takahashi et al. *2010) A multi-valued operator $T : H \rightarrow H$ with the domain $D(T)=\{x \in H : Tx\ne 0\}$ and the range $R(T) = \{Tx : x \in D(T)\}$ is said to be monotone if for $x_1 ,x_2 \in D(T), y_1, y_2 \in R(T)$, the following inequality holds $\langle x_1-x_2, y_1-y_2 \rangle \ge 0$.

### **Definition 2**

(*Takahashi et al. *2010) A monotone operator *T* is said to be maximal if its graph $G(T)=\{(x, y) : y \in Tx\}$ is not properly contained in the graph of any other monotone operator.

### **Definition 3**

(*Takahashi et al. *2010) Let *I* denote the identity operator on *H* and $T : H\rightarrow H$ be a maximal monotone operator. For each $\lambda > 0$, a nonexpansive single-valued mapping $J_\lambda =(I-\lambda A)^{-1}$ is called the resolvent of *T*.

And it is known that $T^{-1}(0)=F(J_\lambda )$ for all $\lambda >0$ and $J_\lambda$ is firmly nonexpansive.

Three classical iteration processes are often used to approximate a fixed point of a nonexpansive mapping. The first one was introduced in 1953 by Mann (1953) and is well known as Manns iteration process defined as follows:

2 $$\begin{aligned} {\left\{ \begin{array}{ll} x_0 \quad chosen \ arbitrarily,\\ x_{n+1}=\alpha _nx_n+(1-\alpha _n)Tx_n, \quad n\ge 0, \end{array}\right. } \end{aligned}$$

where the sequence $\{\alpha _n\}$ is chosen in [0,1]. Fourteen years later, Halpern (1967) proposed the new innovation iteration process which resembled Manns iteration (2). It is defined by

3 $$\begin{aligned} {\left\{ \begin{array}{ll} x_0 \quad chosen \ arbitrarily,\\ x_{n+1}=\alpha _nu+(1-\alpha _n)Tx_n, \quad n\ge 0, \end{array}\right. } \end{aligned}$$

where the element $u \in C$ is fixed. Seven years later, Ishikawa (1974) enlarged and improved Mann’s iteration (2) to the new iteration method, which is often cited as Ishikawa’s iteration process and defined recursively by

4 $$\begin{aligned} {\left\{ \begin{array}{ll} x_0 \quad chosen \ arbitrarily,\\ y_{n}=\beta _nx_n+(1-\beta _n)Tx_n, \\ x_{n+1}=\alpha _nx_n+(1-\alpha _n)Ty_n, \quad n\ge 0, \end{array}\right. } \end{aligned}$$

where $\{\alpha _n\}$ and $\{\beta _n\}$ are sequences in the interval [0,1].

Moreover, many authors have studied the common solution problem, that is, find a point in a solution set and a fixed (zero) point set of some nonlinear problems; see, for example, Kamimura and Takahashi (2000), Takahashi and Toyoda (2003), Ye and Huang (2011), Cho and Kang (2011), Zegeye and Shahzad (2012), Qin et al. (2010), Lu and Wang (2012), Husain and Gupta (2012), Noor and Huang (2007), Qin et al. (2009), Kim and Tuyen (2011), Wei and Shi (2012), Qin et al. (2010), Qin et al. (2008), He et al. (2011), Wu and Liu (2012), Qin and Su (2007), Abdel-Salam and Al-Khaled (2012), Qin et al. (2010), Zegeye et al. (2012) and the references therein. In Kamimura and Takahashi (2000), in the framework of real Hilbert spaces, Kamimura and Takahashi investigated the problem of finding zero points of a maximal monotone operator by considering the following iterative algorithm:

5 $$\begin{aligned} x_0 \in H, \quad x_{n+1}=\alpha _n x_n+(1-\alpha _n)J_{\lambda _n}x_n, \quad n=0,1,2, \cdot \cdot \cdot \end{aligned}$$

where $\{\alpha _n\}$ is a sequence in (0,1), $\{\lambda _n\}$ is a positive sequence, $T : H\rightarrow H$ is a maximal monotone, and $J_{\lambda _n} = (I+\lambda _nT)^{-1}.$ They showed that the sequence $\{x_n\}$ generated in (5) converges weakly to some $z \in T^{-1}(0)$ provided that the control sequence satisfies some restrictions. Further, using this result, they also investigated the case that $T=\partial f,$ where $f :H\rightarrow H$ is a proper lower semicontinuous convex function.

Takahashi and Toyoda (2003) investigated the problem of finding a common solution of the variational inequality problem (1) and a fixed point problem involving nonexpansive mappings by considering the following iterative algorithm:

6 $$\begin{aligned} x_0 \in C, \quad x_{n+1}=\alpha _nx_n+(1-\alpha _n)SP_C(x_n-\lambda _nAx_n), \quad \forall n\ge 0, \end{aligned}$$

where $\{\alpha _n\}$ is a sequence in (0,1), $\{\lambda _n\}$ is a positive sequence, $S: C\rightarrow C$ is a nonexpansive mapping, and $A: C\rightarrow H$ is an inverse-strongly monotone mapping. They showed that the sequence $\{x_n\}$ generated in (6) converges weakly to some $z \in VI(C,A) \cap F(S)$ provided that the control sequence satisfies some restrictions.

Hecai (2013) studied the common solution for two monotone operators and a quasi-nonexpansive mapping in the framework of Hilbert spaces. The aim of this paper is to investigate hybrid algorithm for a common zero point of the sum of two monotone operators which is also a fixed point of a family of countable quasi-nonexpansive mappings. We point out two incorrect justifications in the proof of Theorem 2.1 in paper Hecai (2013). Further, we modify and generalize the results of Hecai’s paper, in which only a quasi-nonexpansive mapping was considered. In addition, two family of countable quasi-nonexpansive mappings with uniform closeness examples are provided to demonstrate our results. Finally, we apply the results to variational inequalities.

To obtain our main results in this paper, we need the following lemmas and definitions.

Let *C* be a nonempty, closed, and convex subset of *H*. Let $\{S_n\}_{n=1}^{\infty }: C\rightarrow C$ be a sequence of mappings of *C* into *C* such that $\cap _{n=1}^{\infty }F(S_n)$ is nonempty. Then $\{S_n\}_{n=1}^{\infty }$ is said to be *uniformly closed*, if $p\in \cap _{n=1}^{\infty }F(S_n)$, whenever $\{x_n\}\subset C$ converges strongly to *p* and $\Vert x_n-S_nx_n\Vert \rightarrow 0$ as $n\rightarrow \infty .$

### **Lemma 4**

(Aoyama et al. 2007) *LetCbe a nonempty, closed, and convex subset of*$H,\;A: C\rightarrow H$*be a mapping, and*$B: H\rightarrow 2^H$*be a maximal monotone operator. Then*$F(J_r(I-\lambda A))=(A+B)^{-1}(0).$

Let *C* be a nonempty, closed, and convex subset of *H*,  the projection operator $P_C: E \rightarrow C$ is a map that assigns to an arbitrary point $x \in H$ the minimum point of the norm $\Vert x-y\Vert$, that is, $P_Cx=\overline{x},$ where $\overline{x}$ is a unique solution to the minimization problem

$$\begin{aligned} \Vert \overline{x}-x\Vert = \min _{y \in C}\Vert y-x\Vert . \end{aligned}$$

It is well-known that

$$\begin{aligned} \langle x-P_Cx, P_Cx-y \rangle \ge 0, \quad \forall \ y \in C. \end{aligned}$$

Abdel-Salam and Al-Khaled (2012) proved the following result.

### **Theorem 5**

*LetCbe a nonempty closed convex subset of a real Hilbert space*$H,\;A: C\rightarrow H$*be an*$\alpha$*-inverse-strongly monotone mapping*, $S: C\rightarrow C$*be a quasi-nonexpansive mapping such that*$I-S$*is demiclosed at zero andBbe a maximal monotone operator onHsuch that the domain ofBis included inC*. *Assume that*$F=F(S)\cap (A+B)^{-1}(0)\ne \emptyset .$*Let*$\{\lambda _n\}$*be a positive real number sequence and*$\{\alpha _n\}$*be a real number sequence in [0,1]. Let*$\{x_n\}$*be a sequence ofCgenerated by*

$$\begin{aligned} {\left\{ \begin{array}{ll} x_1\in C,\\ C_1=C, \\ y_n=\alpha _nx_n+(1-\alpha _n)SJ_{r_n}(x_n-\lambda _nAx_n), \\ C_{n+1}=\{z\in C_{n}: \Vert y_n-z\Vert \le \Vert x_n-z\Vert \},\\ x_{n+1}=P_{C_{n+1}}x_1, \quad n\ge 1, \end{array}\right. } \end{aligned}$$

*where*$J_{r_n}=(I+r_nB)^{-1}.$*Suppose that the sequences*$\lambda _n$*and*$\alpha _n$*satisfy the following restrictions:*

$$(a) \ 0 \le \alpha _n \le a < 1;$$

$$(b)\;0<b \le \lambda _n \le c < 2\alpha$$

*Then the sequence*$\{x_n\}$*converges strongly to*$q= P_{F}x_0.$

However, the proof of above Theorem 5 is not correct. First mistake: in page 6, line 16–17, there is a mistake inequality:

$$\begin{aligned} \Vert z_n-p\Vert ^2= \Vert J_{\lambda _n}(x_n-\lambda _n Ax_n)-J_{\lambda _n}(p-\lambda _n Ap)\Vert ^2\\\le \left\langle \left( x_n-\lambda _n Ax_n\right) - \left( p-\lambda _n Ap\right) , z_n-p \right\rangle . \end{aligned}$$

Second mistake: in page 7, -line 5–7, there is a mistake ratiocination:

Since *B* is monotone, we get for any $(u,v) \in B$ that

7 $$\begin{aligned} \left\langle z_n-u, \frac{x_n-z_n}{\lambda _n}-Ax_n-v \right\rangle \ge 0. \end{aligned}$$

Replacing *n* by $n_i$ and letting $i\rightarrow \infty$, we obtain from (7) that

$$\begin{aligned} \langle \omega -u, -A\omega -v \rangle \ge 0. \end{aligned}$$

**Our comments: ** Notice that, the inner product $\langle \cdot , \cdot \rangle$ is not weakly continuous. For example: in Hilbert space $l^2$, let

$$\begin{aligned} x_0&= (1,0,0,0,0, \ldots ),\\ x_1&= (1,1, 0,0,0, \ldots ),\\ x_2&= (1,0, 1,0,0, \ldots ),\\ x_3&= (1,0, 0,1,0, \ldots ),\\&\cdots \cdots . \end{aligned}$$

It is well-known that $\{x_n\}$ converges weakly to $x_0$, but

$$\begin{aligned} \langle x_n,x_n \rangle =2, \quad \langle x_0,x_0 \rangle =1, \end{aligned}$$

so the inner product $\langle x_n,x_n \rangle$ does not converges to $\langle x_0,x_0\rangle$. Therefore,

$$\begin{aligned} \left\langle z_n-u, \frac{x_n-z_n}{\lambda _n}-Ax_n-v \right\rangle \end{aligned}$$

does not converges to

$$\begin{aligned} \langle \omega -u, -A\omega -v \rangle . \end{aligned}$$

In order to modify the iterative algorithm of Theorem 5 and to get more generalized results, we present a new iterative algorithm in this paper. Moreover, the results are applied to variational inequalities.

## Main results

Now we are in the position to give our main results.

### **Theorem 6**

*LetCbe a nonempty closed convex subset of a real Hilbert space*$H,\;A: C\rightarrow H$*be an*$\alpha$*-inverse-strongly monotone mapping, andBbe a maximal monotone operator onHsuch that the domain ofBis included inC*. *Let*$\{S_n\}: C\rightarrow C$*be a family of countable quasi-nonexpansive mappings which are uniformly closed. Assume that*$F=F(S)\cap (A+B)^{-1}(0)\ne \emptyset .$*Let*$\{r_n\}$*be a positive real number sequence and*$\{\alpha _n\}$*be a real number sequence in [0,1). Let*$\{x_n\}$*be a sequence ofCgenerated by*

$$\begin{aligned} {\left\{ \begin{array}{ll} x_1\in C_1=C, \quad chosen \quad arbitrarily, \\ z_n=J_{r_n}(x_n-r_nAx_n),\\ y_n=\alpha _nz_n+(1-\alpha _n)S_nz_n, \\ C_{n+1}= \left\{ z\in C_{n}: \Vert z_n-z\Vert \le \Vert y_n-z\Vert \le \Vert x_n-z\Vert \right\} ,\\ x_{n+1}=P_{C_{n+1}}x_1, \quad n\ge 1, \end{array}\right. } \end{aligned}$$

*where*$J_{r_n}=(I+r_nB)^{-1},\;\liminf _{n\rightarrow \infty } r_n>0,\ r_n \le 2\alpha$*and*$\limsup _{n\rightarrow \infty } \alpha _n<1.$*Then the sequence*$\{x_n\}$*converges strongly to*$q= P_{F}x_0.$

### *Proof*

We divide the proof into six steps.

Step 1. We show that $C_n$ is closed and convex. Notice that $C_1= C$ is closed and convex. Suppose that $C_i$ is closed and convex for some $i\ge 1$. Next we show that $C_{i+1}$ is closed and convex for the same *i*. Since

$$\begin{aligned} C_{i+1}= C_i \cap \left\{ z \in E: \Vert y_i-z\Vert \le \Vert z_i-z\Vert \}\cap \{z \in E: \Vert z_i-z\Vert \le \Vert x_i-z\Vert \right\} \\= C_i \cap \left\{ z \in E: \langle z, y_i-z_i\rangle \le \frac{1}{2} \left( \Vert y_i\Vert ^2-\Vert z_i\Vert ^2\right) \right\} \\&\cap \left\{ z \in E: \langle z, z_i-x_i\rangle \le \frac{1}{2} \left( \Vert z_i\Vert ^2-\Vert x_i\Vert ^2\right) \right\} . \end{aligned}$$

It is obvious that

$$\begin{aligned}&\left\{ z \in E: \langle z, y_i-z_i\rangle \le \frac{1}{2} \left( \Vert y_i\Vert ^2-\Vert z_i\Vert ^2 \right) \right\} , \\&\left\{ z \in E: \langle z, z_i-x_i\rangle \le \frac{1}{2} \left( \Vert z_i\Vert ^2-\Vert x_i\Vert ^2 \right) \right\} \end{aligned}$$

are all closed and convex, so $C_{i+1}$ is closed and convex. This shows that $C_n$ is closed and convex for all $n\ge 1$.

Step 2. We show that $F \subset C_n$ for all $n\ge 1$. By the assumption, we see that $F \subset C_1$. Assume that $F \subset C_i$ for some $i\ge 1$. For any $p\in F \subset C_i$, we find from the Lemma that

$$\begin{aligned} p=S_ip=J_{r_i}(p-r_iAp). \end{aligned}$$

Since $J_{r_i}$ is nonexpansive, we have

$$\begin{aligned} \Vert z_i-p\Vert ^2 &= \Vert J_{r_i}(x_i-r_iAx_i)-J_{r_i}(p-r_iAp)\Vert ^2\\&\le \Vert (x_i-r_iAx_i)-(p-r_iAp)\Vert ^2\\&= \Vert (x_i-p)-r_i(Ax_i-Ap)\Vert ^2\\&= \Vert x_i-p\Vert ^2-2r_i \langle x_i-p, Ax_i-Ap \rangle +r^2_i\Vert Ax_i-Ap\Vert ^2\\&\le \Vert x_i-p\Vert ^2-r_i(2\alpha -r_i)\Vert Ax_i-Ap\Vert ^2, \end{aligned}$$

which implies that

8 $$\begin{aligned} \Vert z_i-p\Vert \le \Vert x_i-p\Vert . \end{aligned}$$

On the other hand, we have

9 $$\begin{aligned} \Vert y_i-p\Vert&= \Vert \alpha _iz_i+(1-\alpha _i)S_iz_i-p\Vert \nonumber \\&= \Vert \alpha _i(z_i-p)+(1-\alpha _i)(S_iz_i-p)\Vert \nonumber \\&\le \alpha _i\Vert z_i-p\Vert +(1-\alpha _i)\Vert S_iz_i-p\Vert \nonumber \\&\le \alpha _i\Vert z_i-p\Vert +(1-\alpha _i)\Vert z_i-p\Vert \nonumber \\&= \Vert z_i-p\Vert . \end{aligned}$$

From (8) and (9), we know that $p\in C_{i+1}$. This show $F \subset C_n$ for all $n\ge 1$.

Step 3. We show that $\{x_n\}$ is a Cauchy sequence, so it is convergent in *C*.

Since $x_{n}=P_{C_n}x_0$ and $C_{n+1}\subset C_{n}$, then we obtain

10 $$\begin{aligned} \Vert x_n-x_0\Vert \le \Vert x_{n+1}-x_0\Vert ,\quad {\text{ for }} {\text{ all }}\ n\ge 1. \end{aligned}$$

Therefore $\Vert x_n-x_0\Vert$ is nondecreasing. On the other hand, we have

$$\begin{aligned} \Vert x_n-x_0\Vert =\Vert P_{C_{n}}x_0-x_0\Vert \le \Vert p-x_0\Vert , \end{aligned}$$

for all $p\in F\subset C_{n}$ and for all $n\ge 1.$ Therefore, $\Vert x_n-x_0\Vert$ is also bounded. This together with (10) implies that the limit of $\Vert x_n-x_0\Vert$ exists. Put

11 $$\begin{aligned} \lim _{n\rightarrow \infty }\Vert x_n- x_0\Vert =d. \end{aligned}$$

It is known that for any positive integer *m*,

$$\begin{aligned} \Vert x_{n+m}-x_{n}\Vert ^2&= \Vert x_{n+m}-P_{C_n}x_0\Vert ^2\\&\le \Vert x_{n+m}-x_0\Vert ^2-\Vert P_{C_n}x_0-x_0\Vert ^2\\&= D_f(x_{n+m},x_0)-D_f(x_{n},x_0), \end{aligned}$$

for all $n\ge 1.$ This together with (11) implies that

$$\begin{aligned} \lim _{n\rightarrow \infty }D_f(x_{n+m}, x_{n})=0, \end{aligned}$$

uniformly for all *m*, holds. Therefore, we get that

$$\begin{aligned} \lim _{n\rightarrow \infty }\Vert x_{n+m}- x_{n}\Vert =0, \end{aligned}$$

uniformly for all *m*, holds. Then $\{x_n\}$ is a Cauchy sequence, hence there exists a point $p\in C$ such that $x_n\rightarrow p$.

Step 4. We prove that the limit of $\{x_n\}$ belongs to *F*.

Let $\lim _{n\rightarrow \infty }x_n=q$. Sine $x_{n+1}\in C_{n+1}$, so we have

12 $$\begin{aligned} \Vert y_n-x_{n+1}\Vert \le \Vert z_n-x_{n+1} \Vert \le \Vert x_n-x_{n+1}\Vert \rightarrow 0, \end{aligned}$$

as $$n\rightarrow \infty .$$ Hence

13 $$\begin{aligned} \lim _{n\rightarrow \infty }y_n=q, \quad \lim _{n\rightarrow \infty }z_n=q. \end{aligned}$$

From

$$\begin{aligned} y_n=\alpha _nz_n+(1-\alpha _n)S_nz_n, \end{aligned}$$

we have that

$$\begin{aligned} \Vert y_n-z_n\Vert =(1-\alpha _n)\Vert S_nz_n-z_n\Vert . \end{aligned}$$

The condition $\limsup _{n\rightarrow \infty }\alpha _n<1$ and (13) imply that

14 $$\begin{aligned} \lim _{n\rightarrow \infty } \Vert S_nz_n-z_n\Vert =0. \end{aligned}$$

Because $\{S_n\}$ is an uniformly closed family of countable quasi-nonexpansive mappings, therefore this together with the (14) implies that $q \in \cap ^{n=1}_{\infty }F(S_n)$.

Step 5. We show that $q \in (A + B)^{-1}(0)$.

Notice that $z_n =J_{r_n}(x_n-r_nAx_n)$. This means that

$$\begin{aligned} x_n-r_nAx_n \in z_n+r_nBz_n, \end{aligned}$$

Actually, that is,

$$\begin{aligned} \frac{x_n-z_n}{r_n} -Ax_n \in Bz_n, \end{aligned}$$

For *B* is monotone, so we get for any $(u, v) \in B$ that

15 $$\begin{aligned} \left\langle z_n-u, \frac{x_n-z_n}{r_n}-Ax_n -v \right\rangle \ge 0. \end{aligned}$$

Letting $n\rightarrow \infty$, we obtain from (15) that

$$\begin{aligned} \langle q-u, -Aq-v \rangle \ge 0. \end{aligned}$$

Since *B* is a maximal monotone operator, so we have $-Aq \in Bq$, that is, $0\in (A+B)(q)$. Hence, $q\in (A + B)^{-1}(0)$. This completes the proof that $q \in F$.

Step 6. We show that $q=P_{F}x_0$.

Observe that $P_Fx_0 \in C_{n+1}$ and $x_{n+1}=P_{C_{n+1}}x_0$, thus we have

$$\begin{aligned} \Vert x_{n+1}-x_0\Vert \le \Vert P_Fx_0-x_0\Vert . \end{aligned}$$

On the other hand, we have

$$\begin{aligned} \Vert x_0-P_Fx_0\Vert \le \Vert x_0-q\Vert =\lim _{n\rightarrow \infty }\Vert x_0-x_{n+1}\Vert \le \Vert x_0-P_Fx_0\Vert . \end{aligned}$$

Since *F* is closed and convex, so the projection $P_{F}x_0$ is unique. Therefore we get that $q=P_{F}x_0$. This completes the proof. $\square$

## Application

In this section, we apply our results to variational inequalities.

Let $$f: H\rightarrow ($$$$- \infty ,+\infty ]$$ be a proper lower semicontinuous convex function. For all $$x\in H,$$ define the subdifferential

$$\begin{aligned} \partial f(x)=\{z\in H: f(x)+\langle y-x,z \rangle \le f(y), \quad \forall y\in H\}. \end{aligned}$$

Then $\partial f$ is a maximal monotone operator of *H* into itself (Noor and Huang 2007). Let *C* be a nonempty closed convex subset of *H* and $i_C$ be the indicator function of *C*,  that is,

$$\begin{aligned} i_C x={\left\{ \begin{array}{ll} 0, \quad x\in C,\\ \infty , \quad x \notin C. \end{array}\right. } \end{aligned}$$

Furthermore, for any $\nu \in C,$ we define the normal cone $N_C(\nu )$ of *C* at $\nu$ as follows:

$$\begin{aligned} N_C{\nu }=\{z\in H: \langle z,y-\nu \rangle \le 0, \quad \forall y\in H\}. \end{aligned}$$

Then $$i_C : H\rightarrow ($$$$-\infty ,$$$$+\infty ]$$ is a proper lower semicontinuous convex function on *H* and $$\partial i_C$$ is a maximal monotone operator. Let $$Jx=(I+\lambda \partial i_C)^{-1}x$$ for any $$\lambda >0$$ and $$x\in H.$$ From $$\partial i_C x = N_Cx$$ and $$x\in C,$$ we get

$$\begin{aligned} \nu =J_{\lambda }x&\Leftrightarrow x\in \nu +\lambda N_C \nu ,\\&\Leftrightarrow \langle x-\nu ,y-\nu \rangle , \quad \forall y\in C, \\&\Leftrightarrow \nu =P_C x, \end{aligned}$$

where $P_C$ is the projection operator from *H* into *C*. In the same way, we can get that $x\in (A + \partial i_C)^{-1}(0) \Leftrightarrow x\in VI(A,C).$ Putting $B = \partial i_C$ in Theorem 6, we can see that $J_{\lambda _n}=P_C.$ Naturally, we can obtain the following consequence.

### **Theorem 7**

*LetCbe a nonempty closed convex subset of a real Hilbert space*$H,\;A: C\rightarrow H$*be an*$\alpha$*-inverse-strongly monotone mapping, and*$S_n: C\rightarrow C$*be a family of countable quasi-nonexpansive mappings which are uniformly closed. Assume that*$F=F(S)\cap VI(C,A)\ne \emptyset .$*Let*$\{r_n\}$*be a positive real number sequence and*$\{\alpha _n\}$*be a real number sequence in [0,1). Let*$\{x_n\}$*be a sequence ofCgenerated by*

$$\begin{aligned} {\left\{ \begin{array}{ll} x_1\in C_1=C, \quad chosen \quad arbitrarily, \\ z_n=P_C(x_n-r_nAx_n),\\ y_n=\alpha _nz_n+(1-\alpha _n)S_nz_n, \\ C_{n+1}=\{z\in C_{n}: \Vert z_n-z\Vert \le \Vert y_n-z\Vert \le \Vert x_n-z\Vert \},\\ x_{n+1}=P_{C_{n+1}}x_1, \quad n\ge 1, \end{array}\right. } \end{aligned}$$

*where*$J_{r_n}=(I+r_nB)^{-1},\;\liminf _{n\rightarrow \infty } r_n>0, \ r_n\le 2\alpha$*and*$\limsup _{n\rightarrow \infty } \alpha _n<1.$*Then the sequence*$\{x_n\}$*converges strongly to*$q= P_{F}x_0.$

Based on Theorem 7, we have the following corollary on variational inequalities.

### **Corollary 8**

*LetCbe a nonempty closed convex subset of a real Hilbert space*$H,\;A: C\rightarrow H$*be an*$\alpha$*-inverse-strongly monotone mapping. Assume that*$F=VI(C,A)\ne \emptyset .$ Let $\{r_n\}$ be a positive real number *sequence. Let*$\{x_n\}$*be a sequence ofCgenerated by*

$$\begin{aligned} {\left\{ \begin{array}{ll} x_1\in C_1=C, \quad chosen \quad arbitrarily, \\ z_n=P_C(x_n-r_nAx_n),\\ C_{n+1}=\{z\in C_{n}: \Vert z_n-z\Vert \le \Vert x_n-z\Vert \},\\ x_{n+1}=P_{C_{n+1}}x_1, \quad n\ge 1, \end{array}\right. } \end{aligned}$$

*where*$J_{r_n}=(I+r_nB)^{-1},$*and*$\liminf _{n\rightarrow \infty } r_n>0, \ r_n\le 2\alpha .$*Then the sequence*$\{x_n\}$*converges strongly to*$q= P_{VI(C,A)}x_0.$

## Examples

Let *H* be a Hilbert space and *C* be a nonempty closed convex and balanced subset of *H*. Let $\{x_n\}$ be a sequence in *C* such that $\Vert x_n\Vert =r>0, \{x_n\}$ converges weakly to $x_0\ne 0$ and $\Vert x_n-x_m\Vert \ge r>0$ for all $n\ne m$. Define a family of countable mappings $\{T_n\}: C\rightarrow C$ as follows

$$\begin{aligned} T_n(x)= \left\{ \begin{array}{ll} \frac{n}{n+1}x_n &\quad if \, x=x_n (\exists \ n\ge 1) ,\\ -x &\quad if \, x\ne x_n (\forall \ n\ge 1). \end{array}\right. \end{aligned}$$

### **Conclusion 9**

$\{T_n\}$*has a unique common fixed point* 0, *i.e.,*$F=\cap _{n=1}^{\infty }F(T_n)=\{0\}$, *for all*$n\ge 0$.

### *Proof*

The conclusion is obvious. $\square$

### **Conclusion 10**

$\{T_n\}$*is a uniformly closed family of countable quasi-nonexpansive mappings*.

### *Proof*

First, we have

$$\begin{aligned} \Vert T_nx-0\Vert = \left\{ \begin{array}{ll} \frac{n}{n+1}\Vert x_n-0\Vert , &\quad if \, x=x_n ,\\ \Vert x-0\Vert \ &\quad if \, x\ne x_n . \end{array}\right. \end{aligned}$$

Therefore

$$\begin{aligned} \Vert T_nx-0\Vert \le \Vert x-0\Vert ^2, \end{aligned}$$

for all $x \in C.$ On the other hand, for any strong convergent sequence $\{z_n\}\subset E$ such that $z_n\rightarrow z_0$ and $\Vert z_n-T_nz_n\Vert \rightarrow 0$ as $n\rightarrow \infty$, it is easy to see that there exists sufficiently large nature number *N* such that $z_n\ne x_m$, for any $n, m >N$. Then $Tz_n=-z_n$ for $n>N.$ It follows from $\Vert z_n-T_nz_n\Vert \rightarrow 0$ that $2z_n\rightarrow 0.$ Hence $z_n\rightarrow z_0=0,$ that is $z_0 \in F$. $\square$

### *Example 11*

Let $$E=l^2$$, where

$$\begin{aligned} l^2=& {} \left\{ \xi =(\xi _1, \xi _2, \xi _3, \ldots , \xi _n,\ldots ): \sum _{n=1}^{\infty }|x_n|^2<\infty \right\} ,\\ \Vert \xi \Vert= & {} \left( \sum _{n=1}^{\infty }|\xi _n|^2\right) ^\frac{1}{2}, \ \quad\forall \ \xi \in l^2,\\ \langle \xi , \eta \rangle= & {} \sum _{n=1}^{\infty }\xi _n\eta _n, \ \forall \ \xi =(\xi _1,\xi _2,\xi _3,\ldots ,\xi _n,\ldots ), \ \eta =(\eta _1,\eta _2,\eta _3,\ldots ,\eta _n\ldots )\in l^2. \end{aligned}$$

Let $\{x_n\}\subset E$ be a sequence defined by

$$\begin{aligned} x_0&= (1, 0, 0,0,\ldots ),\\ x_1&= (1, 1, 0,0,\ldots ),\\ x_2&= (1, 0, 1, 0,0,\ldots ),\\ x_3&= (1, 0, 0, 1, 0,0,\ldots ),\\ .......&...............................\\ x_n&= (\xi _{n,1}, \xi _{n,2}, \xi _{n,3},\ldots , \xi _{n,k},\ldots )\\ .......&............................... ,\\ \end{aligned}$$

where

$$\begin{aligned} \xi _{n,k}= \left\{ \begin{array}{ll} 1 &\quad if \, k=1, \ n+1 ,\\ 0 &\quad if \, k\ne 1, k\ne n+1, \end{array} \right. \end{aligned}$$

for all $n\ge 1$. It is well-known that $\Vert x_n\Vert =\sqrt{2}, \ \forall n\ge 1$ and $\{x_n\}$ converges weakly to $x_0.$ Define a countable family of mappings $T_n: E\rightarrow E$ as follows

$$\begin{aligned} T_n(x)= \left\{ \begin{array}{ll} \frac{n}{n+1}x_n &{} \quad if \, x=x_n ,\\ -x &{} \quad if \, x\ne x_n , \end{array} \right. \end{aligned}$$

for all $n\ge 0$. By using Conclusion 9 and 10, $\{T_n\}$ is a uniformly closed family of countable quasi-nonexpansive mappings.

### *Example 12*

Let $$E=L^p[0,1]\;(1<p<$$+$$\infty )$$ and

$$\begin{aligned} x_n=1-\frac{1}{2^n}, \ n=1,2,3, \cdot \cdot \cdot \ . \end{aligned}$$

Define a sequence of functions in $L^p[0,1]$ as the following expression

$$\begin{aligned} f_n(x)= {\left\{ \begin{array}{ll} \frac{2}{x_{n+1}-x_n}\ \quad if \quad x_n\le x< \frac{x_{n+1}+x_n}{2} ,\\ \frac{-2}{x_{n+1}-x_n} \ \quad if \quad \frac{x_{n+1}+x_n}{2} \le x < x_{n+1}\\ 0 \qquad \qquad \qquad otherwise \end{array}\right. } \end{aligned}$$

for all $n\ge 1$. Firstly, we can see for any $x \in [0,1]$ that

16 $$\begin{aligned} \int ^{x}_{0}f_n(t)dt\rightarrow 0= \int ^{x}_{0}f_0(t)dt, \end{aligned}$$

where $f_0(x)\equiv 0$. It is well-known that the above relation (16) is equivalent to $\{f_n(x)\}$ converges weakly to $f_0(x)$ in uniformly smooth Banach space $L^p[0,1] (1<p<$+$\infty )$. On the other hand, for any $n\ne m$, we have

$$\begin{aligned} \Vert f_n-f_m\Vert= & {} \left( \int ^{1}_{0}|f_n\left( x\right) -f_m\left( x\right) |^pdx\right) ^{\frac{1}{p}}\\= & {} \left( \int ^{x_{n+1}}_{x_n}|f_n\left( x\right) -f_m\left( x\right) |^pdx+\int ^{x_{m+1}}_{x_m}|f_n\left( x\right) -f_m\left( x\right) |^pdx\right) ^{\frac{1}{p}} \\= & {} \left( \int ^{x_{n+1}}_{x_n}|f_n\left( x\right) |^pdx+\int ^{x_{m+1}}_{x_m}|f_m\left( x\right) |^pdx\right) ^{\frac{1}{p}}\\= & {} \left( \left( \frac{2}{x_{n+1}-x_n}\right) ^p \left( x_{n+1}-x_n\right) +\left( \frac{2}{x_{m+1}-x_m}\right) ^p\left( x_{m+1}-x_m\right) \right) ^{\frac{1}{p}} \\= & {} \left( \frac{2^p}{\left( x_{n+1}-x_n \right) ^{p-1}} +\frac{2^p}{\left( x_{m+1}-x_m \right) ^{p-1}}\right) ^{\frac{1}{p}}\\\ge & {} \left( 2^p +2^p\right) ^{\frac{1}{p}}>0. \end{aligned}$$

Let

$$\begin{aligned} u_n(x)=f_n(x)+1, \quad \forall \ n\ge 1. \end{aligned}$$

It is obvious that $u_n$ converges weakly to $u_0(x)\equiv 1$ and

17 $$\begin{aligned} \Vert u_n-u_m\Vert =\Vert f_n-f_m\Vert \ge (2^p +2^p)^{\frac{1}{p}}>0, \quad \forall \ n\ge 1. \end{aligned}$$

Define a mapping $T: E\rightarrow E$ as follows

$$\begin{aligned} T_n(x)= \left\{ \begin{array}{ll} \frac{n}{n+1}u_n &{} if \, x=u_n (\exists \ n\ge 1) ,\\ -x &{} if \, x\ne u_n (\forall \ n\ge 1). \end{array}\right. \end{aligned}$$

Since (17) holds, by using Conclusion 9 and 10, we know that $\{T_n\}$ is a uniformly closed family of countable quasi-nonexpansive mappings.
